# Assessment of knowledge and practice of health workers towards tuberculosis infection control and associated factors in public health facilities of Addis Ababa, Ethiopia: A cross-sectional study

**DOI:** 10.1186/s13690-015-0062-3

**Published:** 2015-03-25

**Authors:** Girma Demissie Gizaw, Zewdie Aderaw Alemu, Kelemu Tilahun Kibret

**Affiliations:** Department of Disease Prevention and Control, Addis Ababa Health Bureau, Addis Ababa, Ethiopia; Department of Public Health, College of Medical and Health Science, Debre Markos University, Debre Markos, Ethiopia; Departments of Public Health, College of Medical and Health Science, Wollega University, Nekemte, Ethiopia

**Keywords:** Knowledge, Practices, Health worker, Tuberculosis infection control

## Abstract

**Background:**

Tuberculosis is the leading causes of mortality among infectious diseases worldwide. The risk of transmission from patients to health workers is doubles that of the general population. The close contact to the infectious case before diagnosis is the major risk for tuberculosis infection. The aim of the study was to assess knowledge and practice of health professionals towards tuberculosis infection control and its associated factors in health facilities of Addis Ababa, Ethiopia.

**Methods:**

A cross-sectional study was conducted from February 29 to April 15/2014 in selected health facilities in Addis Ababa. Five hundred ninety health workers were included in the study. The sample size was assigned to each health facility proportional to their number of health workers. Study subjects were selected from each stratum by simple random sampling technique. Interviewer administered structured questionnaire was used to collect information. Logistic regression was used to identify factors associated with knowledge and practice of health workers towards tuberculosis infection control.

**Result:**

Five hundred eighty two participants with 98.6% response rate were involved in the study. Of these, 36.1% had poor knowledge and 51.7% unsatisfactory practice score towards tuberculosis infection control. Having more than six years working experience in health facility (AOR = 2.51; 95% CI: 1.5-4.1) and tuberculosis related training (AOR = 2.51 95% CI; 1.5, 4.1) were significantly associated with knowledge on tuberculosis infection control. Having experience in tuberculosis clinic (AOR =1.93; 95% CI: 1.12, 3.34) and tuberculosis related training (AOR = 1.48; 95% CI: 1.87, 2.51) were significantly associated with practice on tuberculosis infection control.

**Conclusion:**

One third of health workers had relatively poor knowledge and nearly half of them had unsatisfactory practice on tuberculosis infection control. Tuberculosis training and work experiences in health facility are determinant factor to knowledge. Whereas tuberculosis related training and experience in tuberculosis clinic are predictor to practice. So, training of the health professionals, on job orientations of junior health workers, and farther study including private health workers are recommended.

## Background

Tuberculosis (TB) is the leading causes of mortality among infectious diseases in the world [1]. According to world health organization (WHO) 2013 report, 8.6 million tuberculosis cases and 1.3 million deaths estimated in 2012 globally [2].

In the region of Africa and South East Asia, the prevalence of tuberculosis among people living with human immunodeficiency virus (HIV) is estimated to 303 and 264 cases per 100,000 populations respectively and 75% of total TB deaths occurred in these regions in 2012 [2,3].

More than four million people suffer from active TB and 650,000 deaths occurred every year in Africa. In Sub-Saharan Africa, TB comprises 25% of avoidable adult deaths and the transmission of multi-drug resistant TB (MDR-TB) among HIV-infected individuals in hospitals has been documented with high case-fatality rates [1-4]. The impact of TB on morbidity and mortality among people living with HIV and drug resistant TB is increasing in many African countries [5]. Ethiopia is among the 22 high TB burden countries with 210 cases per 100,000-population TB prevalence and 44 cases per 100,000-mortality rate [2].

Tuberculosis is infectious when it occurs in the lungs or larynx until has three negative acid-fast bacilli sputum smear results after starting anti- tuberculosis treatment [6,7]. The undiagnosed or unsuspected patient with TB disease is the primary risk to health-care workers and to the general population. The transmission risk of mycobacterium tuberculosis from patients to health-care workers is a neglected problem in many low- and middle-income countries [3]. The major risks for TB infection are through close contact to the infectious case before diagnosis. Household members of persons with infectious TB and health workers are at high risk of becoming infected with TB [2]. Drug-resistant TB is a growing global problem and is more difficult and expensive to treat and cure. There is often a delay in recognizing drug-resistant TB that can lead to prolonged exposure, which increases TB transmissions. In addition, patients with drug-resistant TB remain infectious for much longer, even if treatment is initiated [3,7].

The global efforts to control TB was strengthened in 1991, and recognized as a major global public health problem. Two targets for TB control were established; 70% of case detection rate and 85% of cure rate by the year 2000. These two targets were embedded within the directly observed therapy – short course (DOTS) strategy launched by WHO in 1994, and subsequently endorsed by the WHO STOP TB Strategy in 2006 [2,3].

To prevent TB transmission in health care settings, it is recommended using infection control measures such as use of protective masks by health care workers, administrative controls and environmental measures like natural and mechanical ventilation systems [3,6,8,9]. The infection control measures need to promote early identification of cases, adherence to treatment and implementation of proper TB infection control measures in the household and health facility. Health workers also have to educate TB patients and the community about adequate ventilation [10,11]. Regardless of the knowledge of health workers, there is poor practice on tuberculosis infection control in the health facilities [9,12-15]. Studies shown that practice of health workers towards TB infection control (TBIC) associated with working units (working in TB ward, laboratories, general medicine wards and emergency rooms) [8]. Most health workers (71%) are at higher risk for occupational acquisition of TB [9].

In Ethiopia, the effort of controlling tuberculosis began in the early 1960s with the establishment of TB centers and sanatorium in three major urban areas of the country. A standardized TB prevention and control program, incorporating DOTs was started in 1992 [4]. TB case detection rate was very low throughout the country at the beginning of DOTs strategies and progressively increased. According to WHO 2013 report, the best new and relapse case detection rate achieved as, 11% in 1995, 33% in 2000, 48% in 2005, 66% in 2010 and 64% in 2012 [2].

Despite heath workers are at higher risk of TB transmission and they have valuable role in infection control, only few studies in Ethiopia illustrated health workers’ knowledge and practice regarding TBIC [9,11]. As these previous studies included only medical doctors and nurses in hospital and private clinics, it might not represent the situation in various professions and level of health facilities of the city. Therefore, this study assessed the level of knowledge and practice of health workers towards tuberculosis infection control and its associated factors in Addis Ababa public health facilities.

## Methods

This cross sectional study was conducted from February 29 to April 15/2014 in public health facilities of Addis Ababa, capital city of Ethiopia. Administratively, the city is divided in to 10 sub cities and 116 Woredas. The total area of the city is 54,000 hectares [16]. According to the 2013 population estimation, the total population of Addis Ababa is more than 4 million. At the time of this study, there were a total of 13 public hospitals, 70 functional public health centers and a total of 7563 health professionals working in Addis Ababa health facilities [17].

All health professionals who had been working in Addis Ababa public health facilities were the source population. All health professionals who were working in selected health facilities who have qualification of doctors, health officers, nurses, x-ray technician, pharmacy personnel and laboratory personnel were included in the study. Health workers who were seriously ill and on annual leave during data collection were excluded.

### Sample size determination

The sample size was determined by using Epi Info version 3.5.1 software (Center for Disease. Control and Prevention, Atlanta, 2004) by single population proportion formula with the assumptions of 95% confidence level, 5% precision. The sample size was calculated for different variable such as proportion of health professionals who had good knowledge about TB infection control (74.4%), proportion of health workers who had good practice on TBIC (63.3%) and TBIC training as determinant factor to good knowledge (89.8%) from previous study conducted in Bahir Dar [18]. Then we took the largest sample size (357) among these by using proportion of health worker who had good practice on TBIC (63.3%). Taking design effect of 1.5 and considering 10% non-responses rate, the calculated sample size was 590 health workers.

### Sampling procedures

First, all public hospitals and health centers in Addis Ababa city were identified. Then, three hospitals and 15-health center were selected by simple random sampling technique. The sample size (590) was allocated to each selected facility proportional to the size of health professionals who were working during data collection. The health professionals were stratified by their professions in each selected facility. The sampling frame (list of health worker by profession) for each stratum was obtained from human resource of that facility. Finally, sample was selected from each stratum by simple random sampling technique.

### Data collection procedures and quality control

The data were collected by using structured questionnaire, which was translated into Amharic from English, back translated and pre-tested for consistency. The data collection questionnaire was prepared in English by principal investigator from previous studies [13,19-22]. The data collectors were nurses who were not working in selected health facilities. The trained nurses collect data by face-to-face interview from the study subjects. To assure the data quality, pre-test was done and the completeness and consistency of questionnaire was checked at the time of data collection. The principal investigator controlled the overall activities through continuous supervision. All completed questionnaire was examined for completeness and consistency during possessing and analysis.

### Data entry, processing and analysis

Data were entered and cleaned using EPI-info version 3.5.1 and exported to SPSS 20.0 version (IBM Corporation, 2012) for data analysis. Frequencies and percentage of different variables was computed. Odds ratio with 95% confidence interval was computed to assess the presence and degree of association between the dependent versus independent variables. Variable having p-value less than 0.05 at the bi-variable analysis was taken for multivariate analysis. And variables having p -value less than 0.05 in multivariable analysis was consider as statistically significant.

Knowledge was assessed by 13 questions focusing on symptoms, transmission, treatment and prevention of tuberculosis. Giving correct answer earned score of 1 if not score 0. Knowledge scores for individuals were calculated and summed up to give the total knowledge score. Accordingly, 13 points was developed. Then knowledge score was categorized into good and poor score if it is equal to or above the mean and below the mean respectively. Likewise practice was assessed by 11 questions and 6 observational lists with a total of 17 questions. Giving correct answer earned score of 1 if not score 0. Participants who score equal or above the mean are considered had good practice and below the mean had poor practice.

### Ethical considerations

Ethical clearance was obtained from the Institutional Review Board (IRB) of the Debre Markos University, College of Medical and Health Science, from Addis Ababa City Health Bureau Ethics Review committee and IBR of Amanuel Specialized Hospital. Informed written consent was obtained from the individual respondents after they had been thoroughly and truthfully informed about the purpose of the study. The confidentiality was assured for all the information provided through restricting persons who accessed the data and personal identifier were not included on questionnaire.

## Results

### Socio demographic characteristics of the study population

From 590 participants selected, 582 study subjects responded with response rate of 98.6%. In this study, majority of respondents, 60.5% were female. The mean age of the respondents was 29 years with minimum age of 19 and maximum 78 years. Concerning educational status majority of the participants (51.9%) has first degree. Professionally most of the respondents 328 (56.4%) were nurses. Majority of the study participants, 341 (58.6%) had less than three year working experience in health facility. One hundred thirty four (23%) were participated in TB related training (Table 1).

**Table 1:** **Socio demographic characteristics of the study population in public health facilities, Addis Ababa, 2014**

**Variable**	**Characteristics**	**Frequency (N = 582)**	**%**
Age	18–29	383	65.8
	30–39	136	23.4
	>40	63	10.4
Sex	Male	228	39.2
	Female	352	60.5
Marital status	Single	308	52.9
	Married	260	44.7
	Divorced and Widowed	14	2.4
Profession	Physician	35	6
	Nurse	66	56.4
	Health Officer	328	11.3
	Lab personnel	49	8.4
	Pharmacy personnel	45	7.7
	Others*	59	10.1
Currently working unit	OPD	181	31.1
	TB clinic and TB ward	30	5.2
	Laboratory	43	7.4
	Pharmacy	46	7.9
	Triage	24	4.1
	Medical ward	32	5.5
	Others**	226	38.8
Educational status	Diploma	280	48.1
	First degree	289	49.7
	Second degree and above	13	2.2
Service year in health facility	<3 years	341	58.6
	3 -6 years	150	25.8
	>6 years	91	15.6
Experience in TB clinics	Yes	134	23
	No	444	76.3
Year of experience in TB clinic	<1 year	57	57
	1-4 years	37	37
	>4 years	6	6
Have TB training	Yes	134	23
	No	444	76.3
Duration of training	<3 days	23	17.6
	4-6 days	59	45
	7-10 days	35	28.2
	>10 days	12	9.2

OPD = outpatient department, TB = Tuberculosis.

*Midwife, radiology, physiotherapy; **MCH, delivery, EPI, FP, physiotherapy.

### Knowledge of health workers towards tuberculosis infection control in Addis Ababa public health facilities

Knowledge was assessed by 13 questions focusing on symptoms, transmission, treatment and prevention of tuberculosis. The Knowledge score for individuals were calculated and summed up to give the overall knowledge. The mean knowledge score for the respondents was 8.92 with (SD = 1.92). Seven (1.2%) of the respondents were able to answer all the questions correctly while one respondent attained the minimum knowledge score of zero. Almost all, 96.4% of the respondents knew that “the door and windows of a room should be open whenever TB suspected or confirmed patient is in the room”. Artions focusing on symptoms, transmission, treatment and prevention of around 68% of participants could not identify different types of mask. Whereas 39% knew that surgical mask cannot protect the health worker from inhaling mycobacterium tuberculosis containing aerosols. From 582 participant 221(36.1%) had poor knowledge score (Table 2).

**Table 2:** **The distribution of health professionals by their tuberculosis infection control knowledge in public health facilities, Addis Ababa, 2014 (n = 582)**

**Knowledge items**	**Frequency**	**%**
The door and windows of a room should be left open	561	96.4
TB suspected should be separated from the rest of the patient	532	91.4
Health worker should minimize the time a TB patient spend in HF.	460	79
Surgical mask cannot protect the health worker from TB	226	38.8
Respirator can protect the HW from TB	315	54.1
TB pts have to be educated to cover their mouse with a handkerchief.	522	89.7
Every facility should establish an IP committee.	581	99.8
TB suspected patients should get priority.	514	88.3
Regular screening of health worker for TB is one of TBIC measures	500	85.9
Fans can be used to reduced TB transmission in TB ward	411	70.6
TB cannot transmitted from person to person by blood contact	502	86.2
Sputum microscopy is the most effective tools for the diagnosis of TB	444	76.7
Health workers identify different masks	188	32.3
Overall knowledge level		
Poor knowledge	221	36.1
Good knowledge	361	63.9

### Determinant factors for knowledge of health workers on tuberculosis infection control

To identify independent predictors of good knowledge, a multivariate logistic regression model was fitted with the variables having a p-value < 0.05 in the bi-variable logistic regression analysis. Accordingly, some variables were remained independent predictors for having good knowledge after controlling other factors. From these, being trained on tuberculosis infection control is significantly associated to good knowledge (adjusted odd ratio (AOR) = 2.41; 95% CI: 1.33, 4.36). Similarly, health workers who have more than six year working experience in health facility are two times more likely be knowledgeable compared to those who had less than three year experience (AOR = 1.97; 95% CI:1.10, 3.5). This study shows that health worker who had first degree and above were 1.49 times more knowledgeable compared to diploma level (AOR = 1.49; 95% CI: 1.47, 2.19) (Table 3).

**Table 3:** **Factors associated with health workers knowledge on tuberculosis infection control, Addis Ababa, May 2014**

**Variable**	**Category**	**Level of knowledge**		**COR (95% CI)**	**AOR (95% CI)**
		**Good**	**Poor**		
		**Frequency**	**Frequency**		
Sex	Male	85	143	1	
	Female	125	227	1.09 (0.77-1.54)	
Age group	18-29	134	259	1	
	30-39	60	79	0.68 (0.46-1.01)	
	> = 40	16	34	1.10 (0.59-2.06)	
Marital status	Single	108	200	1	
	Married	99	161	0.88 (0.62-1.24)	
	Divorced and Widowed	3	11	0.50 (0.33-8.71)	
Educational status	Diploma	112	168	1	1
	First degree and above	98	204	2.08 (1.64-2.65)*	1.49 (1.47-2.19)**
Profession	Doctor and HO	33	68	1	1
	Nurse	118	210	1.78 (1.42-2.23)*	0.96 (0.44-1.35)
	Laboratory and Pharmacy	37	57	1.54 (1.21-2.33)*	1.08 (0.69-1.69)
	Others^❖^	22	37	1.68 (0.99-2.85)	1.19 (0.68-2.09)
Currently working unit	OPD and triage	82	123	1	1
	TB clinic and TB ward	8	22	2.75 (1.22-6.18)*	1.63 (0.66-4.04)
	Laboratory	17	26	1.53 (0.83-2.82)	1.07 (0.41-2.78)
	Pharmacy	15	31	2.07 (1.12-3.83)*	2.18 (0.78-6.14)
	Medical ward	74	152	1.29 (0.64-2.58)	1.01 (0.46-2.89)
	Others^†^	14	18	2.05 (1.56-2.71)*	1.87 (0.32-2.61)*
Experience in HF in years	<3 years	127	224	1	1
	3.5-6 years	56	94	1.3 (0.84-1.86)	1.03 (0.68-1.56)
	>6 years	27	54	2.3 (1.32-4.1)*	1.77 (1.01-3.51)**
Experience in TB clinic	No	188	189	1	1
	Yes	22	83	2.45 (1.48-4.07)*	1.38 (0.73-2.62)
TB related training	No	183	264	1	1
	Yes	26	108	2.51 (1.5-4.1)*	2.41 (1.33-4.36)**

*Significant association (p < 0.05) crude. **Significant association, (p < 0.05) adjusted.

^❖^x-ray personnel, midwife, and physiotherapy personnel.

^†^(MCH, delivery, EPI, FP, radiography, physiotherapy, dressing, injection).

OPD = out patients department, TB = Tuberculosis, HO = health officer.

### Practice of health worker towards tuberculosis infection control

There were 11 questions and 6 observational lists in which each response had one point with a total of 17 marks. Participants who scored equal or above the mean are considered as had good practice. The mean score was 10.33 (SD = 3.07). The range of respondents' practice scores was 1– 17.

In this study, 94% of health workers were opening the window whenever TB suspected or confirmed patient is in the room. However, only 53% of the respondents had opened the window at the time of data collection. Around two third, 62.4% of the respondents were always follow TB treatment guideline to manage new-smear positive case while 20.6% of them used sometimes and 17% were never follow the guideline. Majority, 93.1% of the participant educate TB suspected or confirmed patients on cough etiquette (covering of mouse while coughing, no spitting on the floor, etc).

Regarding personal protective, nearly half, 50.2% of participants use respirator whenever they are approaching TB suspected patient. Only 21.3% of the respondent had surgical mask for TB patient and 17.9% had N-95 mask for health workers or patient supporters (Table 4).

**Table 4:** **The distribution of health professionals by their tuberculosis infection control practice in public health facilities, Addis Ababa, 2014 (n = 582)**

**Practice item**	**Always N (%)**	**Sometimes N (%)**	**Never N (%)**
Facilities leaders monitor and evaluate HWs on TBIC.	156 (26.8)	226 (38.8)	199 (34.2)
Follow TB treatment guideline to treat smear positive pt.	363 (62.4)	119 (20.4)	99 (17.2)
Opening window when TB suspected pts is in the room.	473 (81.3)	78 (13.4)	31 (5.3)
Using mask when approaching TB suspected patient.	292 (50.2)	170 (29.2)	120 (20.6)
giving priority patients coughing in waiting area	404 (69.4)	111 (19.1)	67 (11.5)
Educating TB suspected pts how to cough and sneezing.	470 (80.8)	71 (12.2)	41 (7)
Proper use of fan if available.	382 (65.9)	94 (16.2)	104 (17.9)
Health worker screening for TB after contact with TB pts	417 (71.9)	106 (18.3)	57 (9.8)
Availability of designated sputum produced area for TB pts	297 (51.1)	136 (23.4)	148 (25.5)
Use AFB as diagnostic tools for TB suspected pts.	401 (69)	112 (19.3)	68 (11.7)
Check if mask is airtight and does not allow.	311 (53.7)	148 (25.6)	120 (20.7)
Observational checklist	Yes	No	
The room where participant work has cross-ventilated window and door.	310 (53.3)	272 (46.7)	
Windows of the room of participant working was opened during data collection.	311 (53.4)	271 (46.6)	
Surgical mask was available for TB suspected pts.	123 (21.2)	458 (78.8)	
Was there N95 mask available for HWs?	103 (17.8)	476 (82.2)	
Is there TB treatment guideline available?	132 (22.7)	450 (77.3)	
Is there TB prevention poster posted	146 (25.1)	435 (74.9)	
Overall practice	Poor practice	283 (48.6%)	
	Good practice	299 (51.4%)	

### Determinant factors for tuberculosis infection control practice of health workers

To identify independent predictors of good practice a multivariate logistic regression model was fitted with the variables having a p-value < 0.05 in the bi-variate logistic regression analysis. Accordingly, some variables were remained independent predictors for having good practice after controlling other factors. From these factors, health workers who had experience in TB clinic are two times more likely to have good practice than those had not experience (AOR = 1.93; 95% CI: 1.12, 3.34) and tuberculosis related training had statistical significant association with practice (AOR = 1.48; 95% CI: 1.87, 2.51). The health workers who had first degree and above had less likely satisfactory practice compared to diploma (AOR = 0.64; 95% CI: 0.47, 0.88). Despite, knowledge had no significant association with practice (AOR = 1.12; 95% CI: 0.85, 1.49), the study showed significant positive linear correlations between knowledge and practice (r = 0.237 p = 0.001). The positive correlations between knowledge and practice reaffirm the relationship between knowledge and practice with infection control measures even though the correlation is weak in this study (Table 5).

**Table 5:** **Factors associated with health workers practice on tuberculosis infection control, Addis Ababa City, 2014**

		**Good practice**	**Poor practice**	**COR (95% CI)**	**AOR (95% CI)**
		**Frequency**	**Frequency**		
Gender	Male	118	110	1	
	Female	164	188	0.81 (0.58-1.13)	
Age group	18-29	188	205	1	
	30-39	62	77	1.3 (0.86-1.87)	
	> = 40	33	17	1.1 (0.61-1.93)	
Marital status	Single	154	154	1	
	Married	118	142	0.83 (0.59-1.16)	
	Divorced	11	3	3.67 (1.0-13.4)	
Educational status	Diploma	147	133	1	1
	1^st^ degree above	135	166	0.74 (0.535-1.02)	0.64 (0.47-0.88)*
Profession	Doctor	12	23	1	
	Nurse	162	166	0.54 (0.23-1.28)	
	Health officer	35	31	1.01 (0.58-1.76)	
	Laboratory personnel	21	28	1.2 (0.58-2.36)	
	Pharmacy personnel	29	30	0.78 (0.36-1.66)	
	Others^❖^	24	21	1.18 (0.54-2.57)	
Currently working unit	OPD	92	93	1	
	TB clinic	14	12	1.18 (0.517-2.69)	
	Laboratory	18	25	0.728 (0.37-1.42)	
	Pharmacy	24	22	1.10 (0.577-2.10)	
	Triage	12	12	1.10(0.139-7.33)	
	Medical ward	16	16	1.01(0.431-2.37)	
	Others^†^	107	119	0.91(0.62-1.34)	
Experience in HF	<3 years	170	181	1	1
	3.5-6 years	68	82	0.83 (0.60-1.14)	0.79 (0.53-1.17)
	>6 years	45	36	1.30 (0.81-1.94)	0.99 (0.62-1.57)
Had experience in TB clinic	No	215	262	1	1
	Yes	68	37	1.84 (1.23-2.74)*	1.77 (1.02-3.07)**
Have TB training	No	203	244	1	1
	Yes	80	54	1.79 (1.21-2.65)*	1.48 (1.87-2.51)**
Knowledge level	Poor knowledge	92	118	1	1
	Good knowledge	191	181	1.06 (0.86-1.29)	1.12 (0.85-1.49)

*Significant association (p < 0.05) crude. **Significant association, (p < 0.05) adjusted.

^❖^X-ray personnel, midwife, physiotherapy personnel.

^†^(MCH, delivery, EPI, FP, radiography, physiotherapy, dressing, injection).

OPD = outpatient department, HF = health facility.

## Discussion

In this study, nearly two third (63.9%) of the respondents had good overall knowledge about tuberculosis infection control. This finding was lower than the finding of the study conducted in West Gojam which was 74.4% [18] and survey results of hospital staff in South Africa [15]. This finding was also lower than the result from Iraq 90.2% [13]. The discrepancy might be due to the differences in study setup and methods.

The result of this study showed that almost all (96%) of the respondents knew the door and window should be open whenever TB suspected or confirmed patient is in the room. This finding was slightly higher than the study from west Gojam in Ethiopia. Majority, 91.4% of the respondents knew importance of separating TB suspected and 89.7% also knew importance of educating TB patient to cover their mouth with a handkerchief and 88% knew the need of infection control committee. These findings are consistent with recommendation of national TB treatment manual [10] and WHO tuberculosis infection control guideline [7].

Respiratory protection control is the third level of a TB infection control program and consists of the use of protective equipment in situations of a high risk for exposure to TB disease [8]. However, this study shows majority (67.2%) of participants wrongly believed surgical mask can protect health workers from inhaling mycobacterium tuberculosis containing droplets. This result is nearly consistence with the finding from west Gojam [18]. Thirty three percent of the health workers can identify different masks, which is lower than results of the same study conducted in South Africa (89.3%) [15]. This deference might be due to unavailability in the respective health facilities and different data collection methods.

Regarding educational status, the study shows that health workers who had first degree and above were 1.49 times more knowledgeable compared to diploma level. Majority of the respondents (58.6%) had less than three year working experience in health facility. Health workers who had more than six year working experience in health facility were 1.77 times more knowledgeable relative to those who worked less than 3 year. This finding is line with similar study in Kenya but contradict with the result from Bahir Dar [18]. Similarly, health workers who had attained tuberculosis related training had two times more likely to have good knowledge. This is also in line with the finding from west Gojam [18] and from Busia district of Kenya [23].

Practice of the health workers regarding TBIC (48.6%) was not satisfactory. This low practice might be due to low proportion of trained and experienced health workers in respective health facilities. The finding is not consistent with the result (63.3%) of a previous study conducted in selected hospitals of west Gojam of Ethiopia [18]. The discrepancy might be due to differences in sampled health facility level and methods.

Health workers who had first degree and above had less likely satisfactory practice compared to diploma. Even though there is no evidence to show knowledge have significant association with practice, half of health worker who had good knowledge had also good practice while only 43.8% from poor knowledge had good practice. Knowledge score and practice score had linear positive correlation with (r = 0.237 p = 0.001). This positive correlation between knowledge and practice reaffirm that adequate knowledge can lead in good practices.

Only 53.4% window of the room where participant working was open during data collection which is line with previous study in Bahir Dar, 64.9% [18] and the finding of Iraq (43%) [13]. However, this finding is much lower than the finding of similar study on hospital staffs of South Africa (90%) [15]. This inconsistency might be due to facilities structural and training opportunity difference.

During data collection, only 21.3% of the respondent had surgical mask for TB patient. This finding is similar with the result of Bahir Dar (21.1%) [18] better than pervious study finding (8%) in Addis Ababa hospital staffs [9]. On the other hand, only 17.9% N-95 mask were available for health workers or patient supporters. This variation might attribute to supply difference in the study area.

The result of this study indicated that being trained on tuberculosis infection control was 1.4 times more likely to have good practice. This is supported by WHO recommendation such as training of health workers on respirator protection is one measure of risk reduction [8]. This finding is in line with the finding of Bahir Dar (18%) [18]. Experience in TB clinic has also significant association with good practice.

This study has the following limitations: Since the study design used is cross-sectional, the finding regard to factors associated with knowledge and practice might not be strong so that the readers should use the findings cautiously. This study didn’t include other health workers such as patient supporters and cleaners. Lastly although it was expected that participants would answer questions honestly, the presence of the researcher might have influenced them in such a way that they might have answered in a manner that they perceived as correct. Some respondents might give the answers that they thought the researcher was looking for and thus was not their true response (social desire bias).

## Conclusions

Generally, the results of this study revealed that significantly high proportion of health worker had relatively poor over all knowledge. Around three fifth of health workers wrongly believed surgical mask can protect health worker from inhaling mycobacterium containing aerosols and two third of participants could not differentiate surgical mask from N95 and face mask.

More than half of health professionals had relatively unsatisfactory practice towards tuberculosis infection control. Low proportion of the participants were trained on tuberculosis infection control. Only half of the rooms, where health workers are working, had cross ventilated windows.

Educational level (first degree and above), experience in health facility and TB related training are significantly associated with good knowledge whereas experience in TB clinic and TB related training are the independent determinant factor to practice.

Health workers who had first degree and above had more likely to have good knowledge but less likely to have satisfactory practice than those had diploma. The knowledge is not significantly associated with practice.

Training of the health professionals with emphasis of practical aspect is vital to strengthen the implementation of tuberculosis infection control activities. Give on job training for junior staff is important to improve tuberculosis infection control practice. Further study including non-professional health worker, private health facility and managers is recommended. Using focus group discussion or in-depth interview to find out the attitude of health worker towards TBIC is important.

## Abbreviations

AOR: Adjusted odd ratio
BSc: Bachelor Degree of Science
COR: Crude ratio
DOTS: Directly observed treatment, short course
MDR-TB: Multidrug resistant tuberculosis
N95: Airtight personal respiratory protection mask
TB: Tuberculosis
TBIC: Tuberculosis infection control
WHO: World Health Organization
